# TRPM7-like channels are functionally expressed in oocytes and modulate post-fertilization embryo development in mouse

**DOI:** 10.1038/srep34236

**Published:** 2016-09-29

**Authors:** Ingrid Carvacho, Goli Ardestani, Hoi Chang Lee, Kaitlyn McGarvey, Rafael A. Fissore, Karin Lykke-Hartmann

**Affiliations:** 1 Department of Biomedicine, Aarhus University, DK-8000 Aarhus C, Denmark; 2 Centre for Membrane Pumps in Cells and Disease-PUMPKIN, Danish National Research Foundation, Aarhus University, Department of Molecular Biology and Genetics, DK-8000 Aarhus C, Denmark; 3 Department of Biology and Chemistry, Faculty of Basic Sciences, Universidad Católica del Maule, 3480112 Talca, Chile; 4 Department of Veterinary and Animal Sciences, University of Massachusetts, Amherst, MA 01003, USA; 5 Aarhus Institute of Advanced Studies (AIAS), Aarhus University, DK-8000 Aarhus C, Denmark

## Abstract

The Transient Receptor Potential (TRP) channels are a family of cationic ion channels widely distributed in mammalian tissues. In general, the global genetic disruption of individual TRP channels result in phenotypes associated with impairment of a particular tissue and/or organ function. An exception is the genetic ablation of the TRP channel TRPM7, which results in early embryonic lethality. Nevertheless, the function of TRPM7 in oocytes, eggs and pre-implantation embryos remains unknown. Here, we described an outward rectifying non-selective current mediated by a TRP ion channel in immature oocytes (germinal vesicle stage), matured oocytes (metaphase II eggs) and 2-cell stage embryos. The current is activated by specific agonists and inhibited by distinct blockers consistent with the functional expression of TRPM7 channels. We demonstrated that the TRPM7-like channels are homo-tetramers and their activation mediates calcium influx in oocytes and eggs, which is fundamental to support fertilization and egg activation. Lastly, we showed that pharmacological inhibition of the channel function delays pre-implantation embryo development and reduces progression to the blastocyst stage. Our data demonstrate functional expression of TRPM7-like channels in mouse oocytes, eggs and embryos that may play an essential role in the initiation of embryo development.

Following the fusion of a sperm with a mature oocyte, hereafter referred as egg, fertilization triggers initiation of embryo development whose first step is the egg activation. Prior to maturation, immature oocytes are arrested in prophase I of meiosis I, the germinal vesicle stage (GV). Oocytes remain arrested at this stage until puberty, when following the establishment of regular cycles and a surge of luteinizing hormone (LH), they resume meiosis (oocyte maturation), in preparation for fertilization. During maturation, oocytes undergo GV Breakdown, GVBD, complete meiosis I, and re-arrest at the metaphase II of meiosis II (MII stage), which is the stage of ovulation and fertilization^1^. Egg activation is widely thought to be triggered by increases in the intracellular concentrations of free Calcium ([Ca^2+^]_i_), calcium oscillations, which are induced by release of the fertilizing spermatozoon’s “sperm factor”, identified as phospholipase C zeta 1 (PLC ζ)^2^,^3^. The [Ca^2+^]_i_ oscillations initiated by the sperm last several hours and their number, amplitude and frequency impact the developmental competence of the zygote^4^. Despite the intracellular nature of the [Ca^2+^]_i_ oscillations, Ca^2+^ influx from the surrounding media is required to support these oscillations^5^,^6^. Indeed, studies in the mouse have shown that [Ca^2+^]_i_ oscillations cease or show reduced frequency in the absence of extracellular Ca^2+^, which results in depletion of the internal Ca^2+^ stores^5^ and preclude egg activation and initiation of development^7^. Ca^2+^ influx is also required during maturation, as the oocyte’s internal Ca^2+^ stores dramatically increase in content during this process^8^. Nevertheless, Ca^2+^ influx is not required for the resumption of meiosis, although organization of the first spindle and release of the first polar body are dependent on extracellular Ca^2+^ and its influx^9^. Thus, despite a pivotal role for Ca^2+^ influx both during oocyte maturation and egg activation, the molecular identity and function of the Ca^2+^-permeant channel(s) that underlie it is unknown.

Several channels have been proposed to mediate Ca^2+^ influx during maturation and fertilization in mammalian oocytes. Voltage-activated Ca^2+^ (CaV) channels have been reported to be expressed in oocytes of different species^10^, including the mouse^11^, where they recently have been shown to contribute to the increase in intracellular Ca^2+^ store contents during oocyte maturation^12^. Despite this contribution, *Cacnah*^−/−^ female mice showed only a slight reduction in litter size^12^ suggesting that these channels are dispensable for fertility. The Calcium Release Activated Current (CRAC) channels (also known as SOCE (Store-Operated Calcium Entry)), were described to be active in mouse GVs, but their function decreases as maturation progresses^8^,^13^. Neither CRAC nor CaVs have been shown to have essential functions during maturation or fertilization^12^,^14^,^15^,^16^.

The transient receptor potential channels, TRPs, are other possible mediators of Ca^2+^ influx in oocytes and eggs. TRP channels are a family of proteins that include ~30 members grouped in six subfamilies. TRP channels are in general cationic non-selective, weakly voltage sensitive, able to respond to different physiological stimuli and have a wide spectrum of physiological functions^17^. A member of the TRP family, TRPV3, was shown to mediate Ca^2+^ influx in mouse eggs. Selective activation of TRPV3 channels provokes Ca^2+^ entry and parthenogenetic egg activation (parthenogenesis), although its physiological function in eggs remains undetermined, as *Trpv3*-deficient (*Trpv3*^−/−^) mice are fertile^15^,^18^.

Most of the TRPs show tissue-specific expression and the generation of genetically modified mice has been helpful to understand their physiological function^17^. The ubiquitously expressed and member of the Melastatin TRP subfamily, TRPM7, is a bifunctional protein, “chanzyme”, with serine/threonine kinase and ion channel activities^19^,^20^. TRPM7 has been shown to activate downstream of protein G-coupled receptors and respond to intracellular and extracellular Mg^2+^, pH, and phosphate inositol diphosphate (PIP_2_)^21^. The cellular functions of TRPM7, like the majority of TRP channels, have been difficult to elucidate in part due to the lack of specific pharmacological tools. Recently, new and specific TRPM7 inhibitors and agonists have been shown to effectively target the channel under physiological conditions. Here, we took advantage of NS8593, a compound initially reported to be a K^+^ channel blocker^22^ and, of the small organic molecule, Naltriben^23^, to study and confirm the expression of TRPM7 channels in oocytes, eggs and embryos. It is worth noting that unlike the lack of dramatic phenotypes observed in most genetically modified mice that target TRP channels, *Trpm7* knock-out mice are embryonic lethal before E7.5 of embryogenesis^24^,^25^, which further supports the need to study TRPM7 expression and function in gametes and early pre-implantation embryos. In this study, our results obtained by a combination of electrophysiological, imaging studies and *in-vitro* embryo culture using TRPM7 antagonists and agonists, show functional expression of the channel in oocytes, eggs and 2-cell embryos, and suggest a role in pre-implantation development, as in the presence of TRPM7 specific inhibitors, development is curtailed or slowed.

## Results

### Strontium oscillations are not mediated by TRPV3 channel in GV oocytes

**Figure 1:**
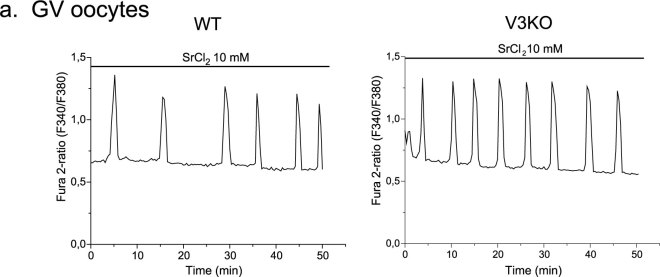
GV oocytes show Sr^2+^-induced Ca^2+^ oscillations that are not mediated by TRPV3 channels. (**a**) Spontaneous oscillations induced by Sr^2+^ at RT in a WT GV oocyte (*left panel*, n = 8, oocytes that show 3 or more oscillations) and in a *Trpv3*^−/−^ oocyte (right panel, n = 6).

Exposure to Sr^2+^ promotes oscillations in both GV oocytes and MII eggs^26^. Nevertheless, in mice, eggs lacking TRPV3 channels are unable to mount Sr^2+^-induced oscillations and undergo egg activation, confirming the identity of the channel responsible for promoting Sr^2+^ influx in eggs^15^. Whether or not GV oocytes from *Trpv3* KO mice are capable of displaying oscillations when exposed to Sr^2+^ is not known. This possibility should not be discounted given that during oocyte maturation new protein expression changes the protein profile of cytoplasmic proteins^27^ as well as of membrane proteins^28^, raising the possibility that different channels mediate Sr^2+^ influx in oocytes vs. eggs. To test the capacity of *Trpv3*^−/−^ GV oocytes to mount Sr^2+^ oscillations, we exposed Wild Type (WT) and *Trpv3*^−/−^ GV oocytes to Sr^2+^ (Fig. 1). In WT oocytes, exposure to Sr^2+^ induced the expected oscillations (Fig. 1a, *left panel*), which were comparable to those observed in *Trpv3*^−/−^oocytes (Fig. 1a, *right panel*). Importantly, the Sr^2+^ induced oscillations in WT and *Trpv3*^−/−^ oocytes were unaffected by temperature, which also rules out TRPV3 as the active channel at GV, as TRPV3 is a temperature-sensitive channel^29^,^30^,^31^ and the measurements here were done at RT. These results support our previous findings showing that functional expression of TRPV3 channels is not detectable at the GV stage and increases during maturation^15^. Therefore, the channel(s) mediating Sr^2+^ influx in WT or *Trpv3*^−/−^ oocytes is not TRPV3.

### Oocytes and eggs express a non-selective cationic conductance that is blocked by external Mg^2+^

**Figure 2:**
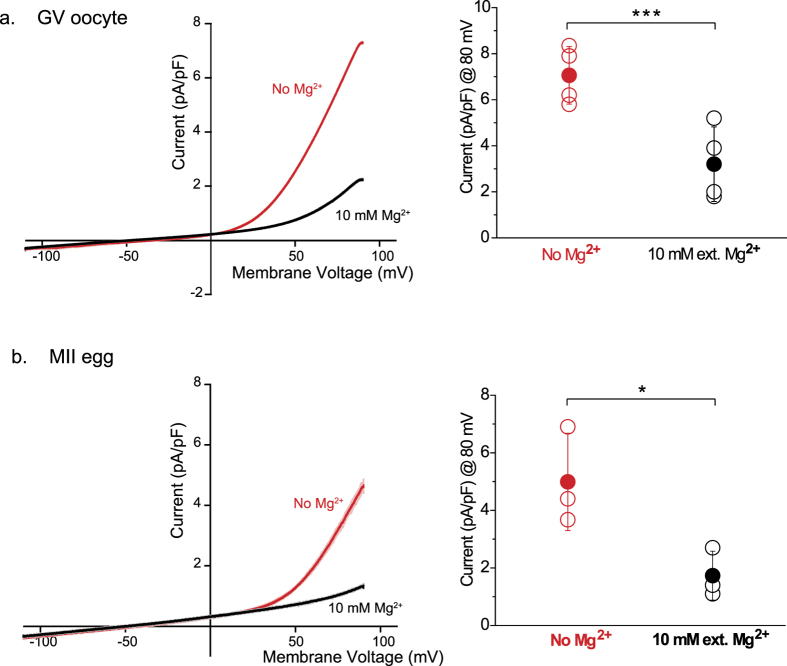
A cationic non-selective channel blocked by external Mg^2+^ is expressed in oocytes and eggs. (**a,b**) Whole-cell voltage clamp recordings from oocytes and eggs. (**a**) *Left panel*: Current evoked from a voltage ramp from −100 to +100 mV in the absence (red trace) and presence of 10 mM external Mg^2+^ (black trace) in GV oocytes. *Right panel*: Averaged current recorded at +80 mV (7, 1 ± 1, 25 pA/pF for recordings in absence of Mg^2+^; n = 4. 3, 2 ± 1, 62 pA/pF for recordings in presence of external Mg^2+^; n = 4). (**b**) Current evoked from a voltage ramp from −100 to +100 mV in the absence (red trace) and presence of 10 mM external Mg^2+^ (black trace) in MII eggs. *Right panel*: Averaged current recorded at +80 mV. (5 ± 1, 7 pA/pF for recordings in absence of Mg^2+^; n = 3. 1, 7 ± 0, 85 pA/pF for recordings in presence of external Mg^2+^; n = 3). ± SD. ***P < 0.001, *P < 0.05.

Extracellular magnesium (Mg^2+^) is a well-known inhibitor of some cationic non-selective channels members of the TRP family, including TRPV3 channels^32^, which are expressed in mouse eggs^15^. Moreover, extracellular and intracellular Mg^2+^ also blocks TRP channels of the Melastatin sub-family, TRPM6 and TRPM7^20^,^33^. We therefore, tested in mouse oocytes and eggs the function of non-cationic channels blocked by this divalent cation. In order to avoid interference, if any, by TRPV3 channels in our electrophysiological recordings, we used *Trpv3*^−/−^ oocytes and eggs. In those gametes and, using internal and external solutions without Mg^2+^, a voltage ramp evoked outwardly rectifying currents with properties characteristics of TRPM7 and/or TRPM6 (Fig. 2a,b, *left panel*, red trace). Addition of 10 mM extracellular Mg^2+^ blocked the TRPM7 or TRPM6-like current in both cells (Fig. 2a,b, *left panel*, black trace), reducing its magnitude (current amplitudes are summarized in Fig. 2a,b, *right panels*). This data shows for the first time the presence of currents that are consistent with functional expression of TRM7- and/or TRPM6-like channels on the plasma membrane (PM) of mouse oocytes and eggs.

### Pharmacological assessment of the current suggests expression of TRPM7 channels in mouse oocytes and eggs

**Figure 3:**
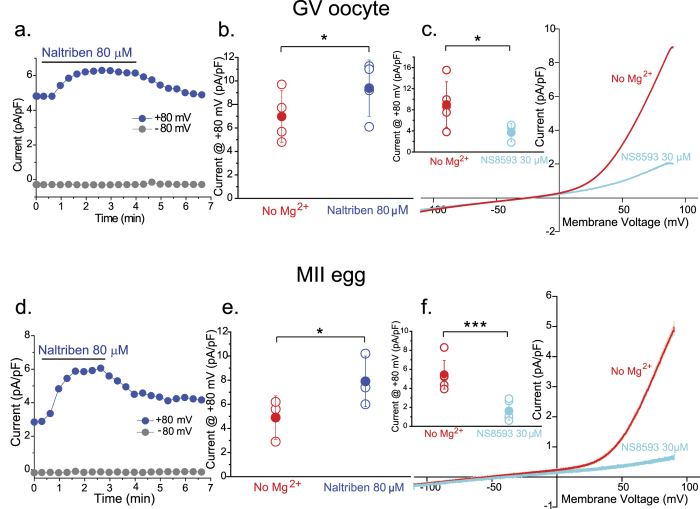
Current responses to drugs targeting TRPM7 channels in mouse oocytes and eggs. (**a,f**) Whole-cell voltage clamp recordings from oocytes (**a–c**) and eggs (**d–f**). (**a**) Whole-cell patch clamp recording in response to 80 μM Naltriben (red bar). (**b**) Averaged current recorded at +80 mV (7 ± 2, 19 pA/pF for recordings in basal conditions (No external Mg^2+^, red symbol); n = 4. 9, 4 ± 2, 4 pA/pF for current responses to 80 μM Naltriben; n = 4). (**c**) Current evoked from a voltage ramp from −100 to +100 mV in response to 30 μM NS8593 (cyan). Insert: Averaged current recorded at +80 mV (9 ± 4, 3 pA/pF for basal responses; n = 5. 3, 7 ± 1, 7 pA/pF for responses to 30 μM NS8593; n = 5). (**d**) Representative trace of a voltage-clamp recording on an egg in response to 80 μM Naltriben (red bar). (**e**) Averaged current recorded at +80 mV (4, 9 ± 1, 8 pA/pF for recordings in basal conditions (No external Mg^2+^, red symbol); n = 3. 7, 9 ± 2, 1 pA/pF for current responses to 80 μM Naltriben; n = 3). (**f**) Current-Voltage (I-V) relation in response to a voltage ramp (−100 mV to +100 mV) in basal conditions (no external Mg^2+^, red trace) and in response to 30 μM NS8593 (cyan). Insert: Averaged current recorded at +80 mV (5, 4 ± 1, 5 pA/pF for basal responses; n = 6. 1, 6 ± 0, 9 pA/pF for responses to 30 μM NS8593; n = 6). ± SD. ***P < 0.001, *P < 0.05.

To test the functional expression of TRPM7 in oocytes and eggs, we made use of a series of compounds with specificity for this channel and determined how they affected its function. TRPM7 current can be blocked by modulators targeting small conductance Ca^2+^-activated K^+^ channels (SK)^22^. NS8593, a gating modulator of the K_Ca_ 2.1–2.3 channels, was recently found to block with higher selectivity over other TRP channels, currents mediated by native and transfected TRPM7 channels^22^. On the other hand, Naltriben, an organic compound, was recently shown to selectively activate TRPM7 channels under physiological conditions^23^. We therefore, took advantage of these pharmacological agents and performed electrophysiological recordings in oocytes and eggs in the absence of intracellular and extracellular Mg^2+^. We also performed these studies in *Trpv3*^−/−^ oocytes and eggs, as some modulators of TRPM7 channels can affect the activity of TRPV3 channels^34^,^35^. In response to a voltage ramp, Naltriben elicited a TRPM7-like current in both oocytes (Fig. 3a,b) and eggs (Fig. 3d,e), and in its presence the average current amplitude was higher than the basal current in both cell types (summarized, Fig. 3b,e). Addition of NS8593 effectively blocked the TRPM7- like current (Fig. 3c,f). Collectively, these results are consistent with functional expression of TRPM7-like channels in mouse oocytes and eggs.

### Naltriben induces Ca^2+^ influx in mouse oocytes and eggs

**Figure 4:**
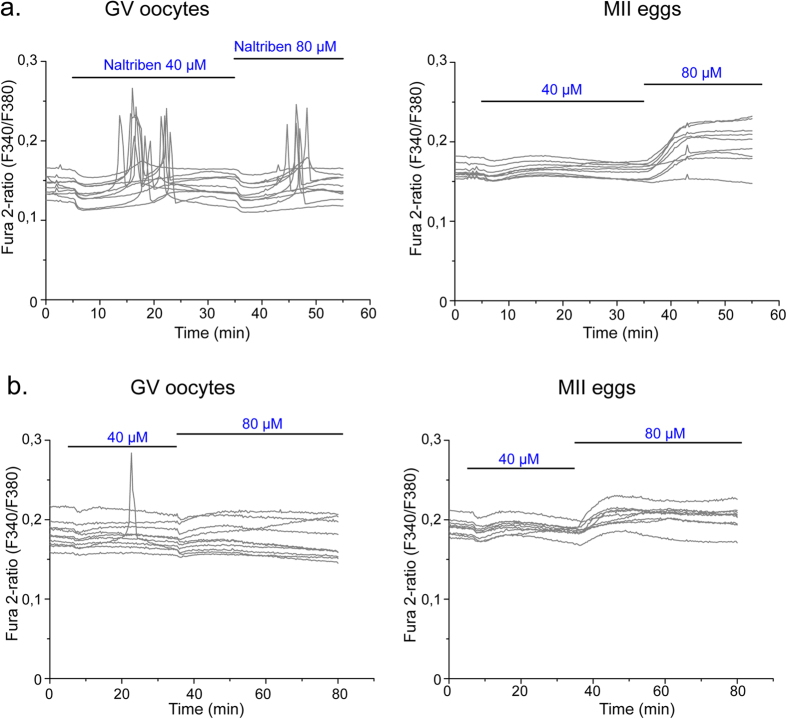
The TRPM7 agonist Naltriben induces [Ca^2+^]_i_ responses in oocytes and eggs. Changes in [Ca^2+^]_i_ were induced by repeated application of 40 and 80 μM Naltriben (black horizontal bars) in Ca^2+^-containing media (*upper panels*) or in Ca^2+^ free-media (*lower panels*). (**a**) *Left panel*, GV oocytes. n = 9. *Right panel*: MII eggs. n = 9. (**b**) *Left panel*, GV oocytes. n = 10. *Right panel*: MII eggs. n = 10. Note that in Ca^2+^ free-media application of Naltriben failed to induce major [Ca^2+^]_i_ changes.

Ca^2+^ influx is required to fill the stores during maturation and to refill them following fertilization^27^, although the channels and/or transporters through which this is accomplished are unknown. To further evaluate the capacity of TRPM7 to mediate Ca^2+^ influx in oocytes and eggs, we tested the [Ca^2+^]_i_ responses induced by the TRPM7-specific activator Naltriben. WT oocytes responded to a wide range of Naltriben concentrations with brief [Ca^2+^]_i_ peaks, which were preceded by a transient decrease in baseline [Ca^2+^]_i_ (Fig. 4a *left panel*). On the other hand, MII eggs required higher concentration of Naltriben and the response seems to be sustained (Fig. 4a, *right panel*). To confirm that [Ca^2+^]_i_ increases induced by Naltriben were caused by Ca^2+^ influx and not due to intracellular off targets effects of the drug, we exposed the cells to Naltriben in media without Ca^2+^. Oocytes and eggs failed to show [Ca^2+^]_i_ increase in response to the agonist in absence of Ca^2+^ in the media (Fig. 4b, *left and right panels*). These results suggest that Naltriben-induced [Ca^2+^]_i_ increases in oocytes and eggs are caused by Ca^2+^ influx mediated by TRPM7 channels.

### NS8593 blocked Ca^2+^ and Sr^2+^ induced oscillations in oocytes but not Sr^2+^-induced oscillations in eggs

**Figure 5:**
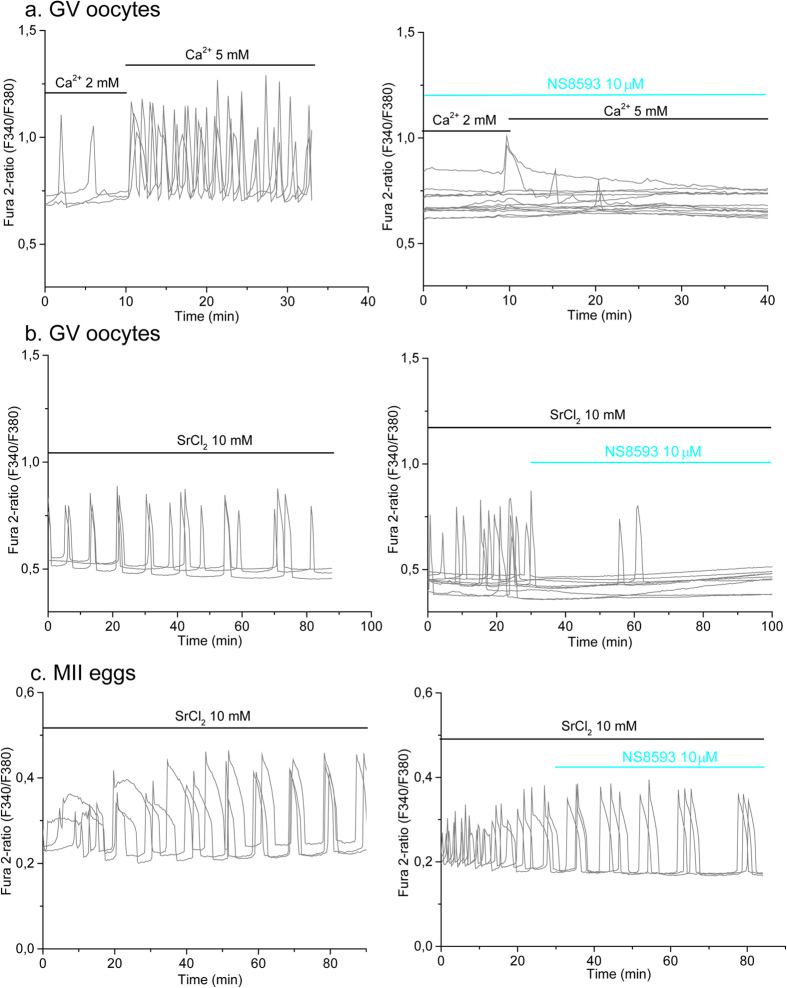
NS8593 blocks [Ca^2+^]_i_ and Sr^2+^ induced oscillations in oocytes. Spontaneous [Ca^2+^]_i_ oscillations were induced and measured at RT in response to increasing concentrations of extracellular Ca^2+^ and 10 mM Sr^2+^ in WT oocytes. (**a**) *Left panel*, [Ca^2+^]_i_ oscillations were evaluated in response to increased extracellular Ca^2+^ (black horizontal bars). Figure shows representative traces for 3 oocytes. n = 9. *Right panel*: Oocytes were exposed to 10 μM NS8593 (cyan bar) and Ca^2+^ oscillations were evaluated after increasing external Ca^2+^ concentration from 2 or 5 mM (black horizontal bars). n = 13. (**b**) *Left panel*: Representative traces for 3 oocytes showing Sr^2+^-induced oscillations. n = 5, oocytes that show 3 or more oscillations. *Right panel*: Sr^2+^-induced oscillations were blocked by exposure to 10 μM NS8593 (cyan bar). n = 9. (**c**) Oscillations induced by Sr^2+^ at 33–35 °C (*left panel*, n = 5. Figure shows representative traces for 3 eggs) are not blocked by 10 μM NS8593 (cyan bar; *right panel,* n = 7. Figure shows representative traces for 3 eggs).

GV oocytes display spontaneous [Ca^2+^]_i_ oscillations which are inhibited by high concentration of Nickel, an unspecific blocker of Ca^2+^ channels^36^. The channel(s) that mediates Ca^2+^ influx supporting these oscillations in oocytes is unknown. As we have shown in this study, TRPM7-like channels expressed in oocytes are able to mediate Ca^2+^ influx. Therefore, to further evaluate the role of TRPM7 channels supporting Ca^2+^ oscillations in oocytes, we tested the efficacy of NS8593 in blocking these [Ca^2+^]_i_ responses. As expected, WT oocytes displayed spontaneous [Ca^2+^]_i_ oscillations in presence of 2 mM extracellular Ca^2+^ (Fig. 5a, *left panel*). The frequency of the oscillations rapidly increased by bringing the external Ca^2+^ concentration to 5 mM (see Fig. 5a, *left panel*), suggesting enhanced Ca^2+^ influx. The addition of NS8593 to the culture media prevented both spontaneous and Ca^2+^ (5 mM) enhanced oscillations (Fig. 5a *right panel*). These results suggest that TRPM7-like channels may underlie [Ca^2+^]_i_ oscillations in GV oocytes.

As previously shown by others and us, external Sr^2+^ induces periodic Ca^2+^ oscillations in oocytes (Fig. 5b *left panel*)^26^ and eggs^15^,^26^ (Fig. 5c *left panel*), although the channel(s) that mediate this influx in GV oocytes remains unknown. We therefore examined if NS8593 could block Sr^2+^ induced oscillations in WT GV oocytes and eggs. We found that whereas addition of NS8593 blocked oscillations in GV oocytes (Fig. 5b *right and left panel*) it was without effect in MII eggs (Fig. 5c *right and left panel*). Our results suggest that a common channel, most likely TRPM7-like channels, mediate Ca^2+^ and Sr^2+^ influx in GV oocytes. In agreement with our previous data^15^, these results also point out that TRPM7 channel does not contribute to the Sr^2+^-induced oscillations observed in eggs, clearly indicating that different channels mediate Sr^2+^ influx in GV oocytes and MII eggs. Finally, our data showed that NS8593 does not inhibit TRPV3 channels.

### TRPM7 homomers and not heteromers with TRPM6 are expressed in oocytes

**Figure 6:**
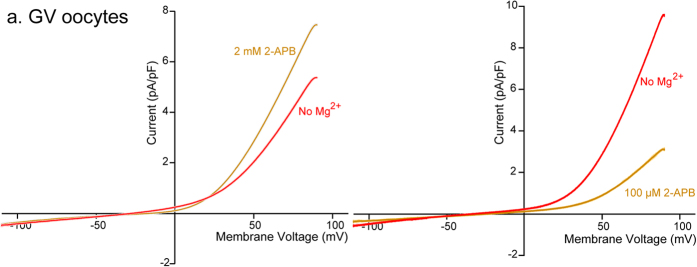
Modulation of native TRPM7 current by 2-APB in oocytes. Whole-cell voltage clamp recordings from oocytes. Current evoked from a voltage ramp from −100 to +100 mV. *Left panel.* 2-APB 2 mM significantly increased TRPM7 current in GV oocytes (n = 4). *Right panel.* 2-APB 100 μM blocked TRPM7 current (n = 4).

Like TRPM7, TRPM6 channels belong to the subfamily Melastatin of TRP channels and, contain an α serine/threonine kinase domain. TRPM6 homo-oligomers show slightly higher permeability to Mg^2+^ than Ca^2+^, and are modulated by Mg^2+^ and ATP^37^. TRPM6 has been shown to form hetero-tetramers with TRPM7^38^, and it was of interest to determine the structural composition of the TRPM-like channels expressed in oocytes and eggs. To accomplish this, we used electrophysiological recordings and 2-APB, which besides being both an unspecific Ca^2+^ channel blocker and an agonist of TRPV3 channels^34^,^39^, it has dual pharmacological effects on TRPM6 and TRPM7 channels; this effect can be used to discriminate whether these channels are assembled in homomeric or heterotetramic configurations^35^. To avoid any contamination of the TRPV3 currents in our recordings^15^,^40^, we performed the electrophysiological recordings in *Trpv3*^−/−^ oocytes (Fig. 6) and eggs (see Supplementary Fig. S1). We used two concentrations of 2-APB to examine the currents in response to a voltage ramp; the low concentration was 100 μM, and the high concentration was 2 mM. The low concentration of 2-APB was expected to block TRPM7 channels but increase currents through TRPM6 homo-oligomers or M6-M7 hetero-tetramers, whereas the high concentration of 2-APB was expected to activate TRPM7 currents^41^. Accordingly, the cationic currents evoked by a voltage ramp in oocytes showed a significant increase when exposed to 2 mM 2-APB (Fig. 6a, *left panel*), whereas exposure to 100 μM 2-APB resulted in a marked inhibition of the current (Fig. 6a, *right panel*). Together, these results suggest that TRPM7 homo-tetramers mediate the current recorded in oocytes and eggs.

### TRPM7 functional expression persists post-fertilization

**Figure 7:**
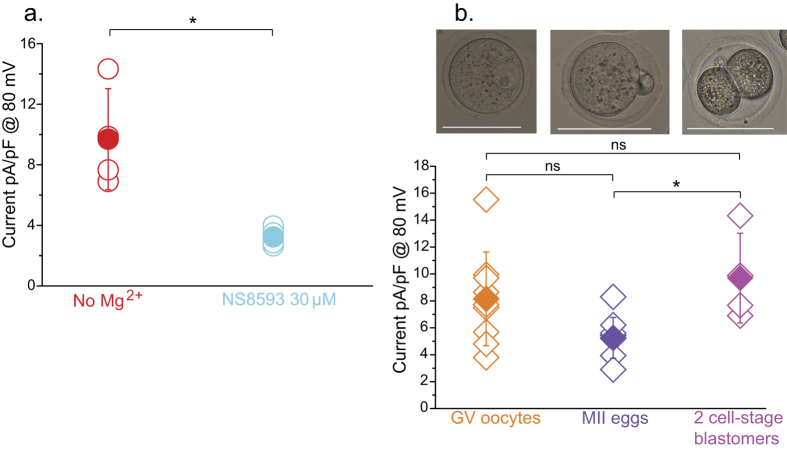
TRPM7 channel is expressed after fertilization. (**a**) Whole-cell patch clamp recordings in blastomeres from 2-cell stage obtained after fertilization. Averaged current at +80 mV is showed. Red symbols: Basal current, 9, 7 ± 3, 3 pA/pF, n = 4. Cyan: Current in response to 30 μM NS8593, 3, 2 ± 0, 6 pA/pF, n = 4. (**b**) Comparison of basal current density between GV oocytes (orange symbols; 8, 1 ± 3, 5 pA/pF, n = 9), MII eggs (blue diamonds; 5, 2 ± 1, 5 pA/pF, n = 9) and 2-cell stage blastomers (violet symbols; 9, 7 ± 3, 3 pA/pF, n = 4) in absence of external Mg^2+^ measured at +80 mV. ± SD. *P < 0.05. Scale bar: 50 μm.

Fertilization triggers the completion of the second meiosis, rendering eggs into zygotes and leading to the first mitotic cleavage and to the 2-cell stage embryo. Ca^2+^ influx is required to support oscillations during the zygote stage, although for how long these channels remain functional in the zygote and/or embryos is not known. Here, we examined the functional expression of TRPM7-like channels 40 h post-fertilization, which corresponds to the late 2-cell stage. To accomplish this, recovered zygotes were allowed to cleave to 2-cell stage embryos and the blastomeres were mechanically separated and treated as units. To detect the presence of the TRPM7-like current in these cells, we evaluated the current response to a voltage ramp using agonists and inhibitors of the channel. We found TRPM7-like channels are expressed in 2-cell blastomeres, as the detected cationic non-selective current was blocked by NS8593 (Fig. 7a). In support of the presence of an active TRPM7 channel in embryos, its agonist Naltriben increased the non-selective current expressed in blastomeres (Supplementary Fig. S2). To assess whether the recorded current was similar to that detected in oocytes and eggs, we compared the density of the currents in 2-cell stage blastomeres to that in oocytes and eggs. Our results show that the TRPM7-like current seemed to experience a decline from the GV to the MII stage, with fertilization inducing an increase in the current that now appears to similar to that detected in GV oocytes (Fig. 7b). Altogether, we interpret our results to mean that TRPM7 channels are functionally expressed in both GV oocytes and eggs and post-fertilization at least until the 2-cell stage embryos.

### TRPM7 blockade prevented pre implantation development in mouse embryos

The finding that the TRPM7-like current is detectable in embryos led us to further evaluate the possible function of TRPM7 on early pre-implantation embryo development. To accomplish this we examined the progression of embryo development in the presence of the TRPM7 inhibitor NS8593. WT zygotes were collected 15 h post hCG injection and exposed to the drug soon after with the notion to avoid any possible effect of NS8593 on sperm entry and on initiation of [Ca^2+^]_i_ oscillations. We found that whereas cleavage to the 2-cell stage was undisturbed in the presence of the inhibitor, subsequent development was greatly affected and progression of the blastocysts was largely prevented (Table 1). To determine when embryo progression was affected, we evaluated embryonic cleavages at shorter intervals. As shown in Table 2 embryos treated with NS8593 cleaved to the 4-cell stage at lower rates (p < 0.05) (Table 2). In addition, and because NS8593 also target SK_Ca_^2+^ channels, we exposed WT zygotes to the K_Ca_^2+^ 2.1–2.3 (SK1, SK2, and SK3 channels) inhibitor apamin^42^, and evaluated embryo development progression in zygotes. Apamin had no effect on development (Supplementary Table S1), excluding SKs channels as NS8593 targets in mouse embryos. Next, we evaluated the impact of on *Trpv3*^−/−^ embryos, as in these embryos there could be possible compensation of channel activities after fertilization. *Trpv3*^−/−^ zygotes were collected 20 h post hCG injection, as we noted that embryos of this strain are unable to proceed past the 2-cell stage if collected 15 h post hCG injection. Similarly to what we observed in WT embryos, *Trpv3*^−/−^ embryos showed delayed developmental progression, which became evident between the 8-cell and morula stages (evaluation at 60 h); progression to the blastocysts stage was also diminished (see Supplementary Table S2). Another specific TRPM7 inhibitor, Waixenicin A (ref. 21), was not used for embryo culture studies, as at the concentrations needed to block oscillations in oocytes and eggs, it caused cell lysis (Supplementary Table S3). In total, our results showed that TRPM7 function is required for normal pre-implantation development, as disruption of the function of the channel impaired development, delaying progression to the morula stage, and inhibiting blastocyst formation.

## Discussion

Here we have examined the functional expression of a member of the TRP family of channels, TRPM7, in mouse oocytes, eggs and 2-cell embryos. Using direct measurements of ion currents by whole-cell voltage-clamp along with Ca^2+^ imaging studies and specific agonists and inhibitors of TRPM7, we found that homo-tetramer TRM7-like channels are functionally expressed in oocytes, eggs and after fertilization. Accordingly, Ca^2+^ influx was enhanced by the TRPM7-specific agonist Naltriben, and spontaneous [Ca^2+^]_i_ (and Sr^2+^) induced oscillations were inhibited by the TRPM7-specific antagonist NS8593. These results suggest a role for TRPM7 in Ca^2+^ influx and homeostasis in oocytes and eggs. Furthermore, our results suggest that this channel is important for pre-implantation development, as *in vitro* culture of embryos in the presence of a specific inhibitor prevented or delayed progression to the blastocyst stage.

GV oocytes undergo spontaneous [Ca^2+^]_i_ oscillations following release from antral follicles^36^. These oscillations are dependent on Ca^2+^ release from intracellular [Ca^2+^]]_i_ stores, but ultimately depend on extracellular Ca^2+^ and Ca^2+^ influx; these oscillations are not required for the GVBD to take place^36^. Oscillations during GV stage can also be observed by replacing external Ca^2+^ with Sr^2+^. Exposure to Sr^2+^ is a well-known method of activation of rodent eggs, as Sr^2+^ induces persistent oscillations in MII eggs that promotes parthenogenesis^26^,^43^,^44^. In this study, we demonstrated that the channel that permeates Sr^2+^ in oocytes is not the same that permeates it in eggs, as whereas *Trpv3*^−/−^ eggs are unable to mount Sr^2+^ oscillations^15^, *Trpv3*^−/−^ GV oocytes do so normally. TRPV3 channels, which are responsible for Sr^2+^ influx in mouse eggs, are temperature sensitive^29^,^30^,^31^, and the oscillations induced by Sr^2+^ in oocytes can occur at RT. Accordingly, Sr^2+^ (and Ca^2+^) induced oscillations in GV oocytes may be mediated by the same channel(s). Consistent with this notion, NS8593 inhibited [Ca^2+^]_i_ and Sr^2+^ oscillations in oocytes, but not in eggs. These results suggest that differential expression exists among oocytes and eggs regarding PM channels that mediate cation influx and efflux. Our results showing functional expression of TRPM7-like channels in GV oocytes suggest this channel, which is permeable to divalent cations including Sr^2+^, is a candidate to mediate Ca^2+^ and Sr^2+^ influx in oocytes.

In this study, we used voltage-clamp and several pharmacological approaches to elucidate the molecular identity of a native cationic non-selective channel(s) present in oocytes, eggs and embryos. Their presence was further supported by the expression of the corresponding transcripts, as previously reported^45^. The channel(s) was blocked by extracellular Mg^2+^, which is consistent with expression of TRPM7^46^. We therefore used a TRPM7- specific inhibitor NS8593, which selectively inhibited native TRPM7-like currents in several cells lines^22^. We also used the TRPM7 agonist Naltriben to determine if it activates similar current to those observed in oocytes and eggs. Naltriben was identified using an aquorin bioluminescence assay to screen small molecules with agonist activity on the TRPM7 channel. Naltriben was determined as the most specific towards TRPM7 among more than 20 compounds that were shown to have activity^23^. The electrophysiological responses and the inhibitory and stimulatory effects of NS8593 and Naltriben, respectively, on the studied currents in oocytes, eggs and embryos are consistent with functional expression of TRPM7-like currents in the germ cells here recorded.

Since TRPM7 has been reported to form heteromers with its homolog TRPM6^38^, we examined whether or not the currents in our cells were mediated by the homo-tetramer TRPM7 channels. To accomplish this we used the non-selective Ca^2+^ blocker 2-APB^47^, which has dual and concentration- dependent actions on TRPM7 currents^35^. Our results showing that 100 μM 2-APB blocked the current in oocytes and eggs, whereas 2 mM worked as an agonist of the channels confirmed the presence of TRPM7 channels in these cells as well as showed that there are of homo-tetrameric composition. Lastly, embryos generated from a *Trpm7* knock-out mouse model died before day 7.5 of embryogenesis (E7.5) showing that TRPM7 is essential to support embryonic development^24^,^25^. Nevertheless, whether TRPM7 function is required at earlier stages of development was not determined. Consequently, and given our findings of functional expression of TRPM7-like channels in oocytes, eggs and 2-cell stage embryos, we investigated whether or not its inhibition could compromise pre-implantation embryo development. To that end, we cultured *in vivo* fertilized zygotes in the presence of NS8593. We noticed that the WT zygotes treated with the inhibitor showed slower cleavage rates prior to reaching the morula stage, and were unable to reach the blastocyst stage. The delayed development was also observed in *Trpv3*^−/−^ embryos, in agreement with the indispensable role of TRPM7 channels suggested by genetic studies^24^. Our results are consistent with a crucial function of TRPM7 channels post-fertilization.

As mentioned, deletion of the mouse *Trpm7* gene, showed it to be the first TRP channel with an essential and non-redundant role in embryogenesis^24^. Later, it was shown that induced global *Trpm7* disruption at E7-E9, was lethal but not after day E14^25^. Interestingly, TRPM7 was found not to be required for the survival of many differentiated cells, but it was indispensable for the survival of pluripotent stem cells and some multipotent stem cells^25^. Consistent with those findings, downregulation of TRPM7 by morpholino injection in *Xenopus* embryos caused severe gastrulation defects, including a short and curved axis and a widely, opened neural tube^48^. Our results showed functional expression of TRPM7 channels before and after fertilization, which suggests a role for TRPM7 in mediating Ca^2+^ influx in these cells and possibly prior to the blastocyst stage. The precise role of TRPM7 on early cleavage state embryos will require more detailed and specific approaches such as knock-down of its expression and function. Our attempts in this direction using siRNA probes and/or a TRPM7 antibody did not cause any developmental phenotype likely due to high levels and stability of maternally contributed TRPM7 and low antibody specificity. These experiments suggest that maternal TRPM7 channels are essential for early development and studies involving oocyte specific *Trpm7*-KO will become crucial to elucidate the function of *Trpm7* during pre-implantation embryo development.

The broad permeability of TRPM7 towards divalent cations raised questions regarding its function in the regulation of cellular Ca^2+^ levels^49^. Initially, TRPM7 was reported as a Mg^2+^ regulated channel^20^, which was confirmed by findings showing inhibition of the TRPM7 current by high intracellular and extracellular Mg^2+^ concentrations; these properties, in fact, were used to differentiate it from CRAC channels^33^. Further, the permeability to and blockade by Mg^2+^ contribute to its essential role in the regulation of cellular Mg^2+^ concentrations, which was later demonstrated in TRPM7-deficient cells whose impaired viability and proliferation were rescued by supplementation with Mg^2+^ extracellular^50^. In addition, Mg^2+^ has been shown to play a fundamental role during development in humans and its deficiency has been linked to birth defects, including spina bifida, defects in fetal growth and development^51^. Moreover, the impaired gastrulation phenotype caused by depletion of TRPM7 in *Xenopus* embryos can be prevented by Mg^2+^ supplementation, by expression of the TRPM7 homologous channel TRPM6, or by the expression of a Mg^2+^ transporter^48^. Additionally, heterozygous animals lacking the α-kinase domain of TRPM7 showed signs of hypomagnesaemia, and stem cells lacking this domain displayed proliferation arrest that was rescued by Mg^2+^ supplementation^52^. The Mg^2+^ requirement for oocyte maturation and egg activation in mammals has not been assessed carefully, although, a recent study showed that lower concentrations of Mg^2+^ *in-vitro* fertilization, IVF, and embryo culture media, 0, 2 mM instead of 1, 2 mM Mg^2+^, improved development to the blastocyst stage^53^. Importantly, the estimated total intracellular Mg^2+^ concentration in somatic cells is between 14 to 20 mM, which is actively maintained by Mg^2+^ transporters and ion channels^54^; the expression of these proteins has not been described in mammalian oocytes and eggs. Thus, it is presently unknown whether TRPM7 contributes to Mg^2+^ regulation in mammalian oocytes and eggs. It is worth noting that in prawn and shrimp oocytes, external Mg^2+^ is the signal that stimulates resumption of meiosis at the time of spawning^55^,^56^, demonstrating that changes in the concentration of this ion are important for the initiation of development. The study of the regulation of Mg^2+^ cellular homeostasis and the role of TRPM7 in Mg^2+^ transport in oocytes needs to be addressed.

Zinc (Zn^2+^) is another ion that permeates TRPM7 channels^35^,^49^. Zn^2+^ has been shown to tightly regulate oocyte maturation as intracellular deficiency in Zn^2+^ provokes arrest in meiotic telophase and fertilization of these oocytes results in inviable embryos^57^. Zn^+^ is also fundamental for cell cycle resumption after fertilization and release of Zn^2+^ to the extracellular media, “Zn^2+^ sparks”, occurs nearly simultaneously with initiation of [Ca^2+^]_i_ oscillations^58^. During oocyte maturation, Zn^2+^-transporters such as ZIP6 and ZIP10 have been shown to be responsible of the accumulation of Zn^2+ ^59, although their function after fertilization remains unclear. TRPM7 conducts Zn^2+^ efficiently^49^, and given that the cytosolic concentration the Zn^2+^ can regulate the binding of the α-kinase domain of TRPM7 to transcription factors containing zinc-fingers, it cannot be ruled out that TRPM7 contributes to gene expression in early embryos. Furthermore, the cleaved kinase domain of TRPM7 can bind components of the Polycomb Repressor Complex 1 (PRC1)^60^, which are proteins associated with transcriptional silencing of genes during oogenesis and embryonic development^61^. Therefore, even when TRPM7 could not be the major mediator of Zn^2+^ influx/efflux in oocytes and embryos, its activity regulating Zn^2+^ homeostasis might have an important role in epigenetic changes during embryo development.

In summary, we demonstrated that homo-tetramers of TRPM7-like channels are expressed in mouse oocytes, eggs and embryos. We showed that TRPM7 is able to mediate Ca^2+^ influx in oocytes, eggs and embryos, suggesting a role of this channel during oocyte maturation, egg activation and early embryo development. Furthermore, we showed that the blockade of the TRPM7 function post-fertilization leads to delayed embryonic development resulting in inhibition of development to the blastocyst stage. The generation of specific oocyte *Trpm7*-KO mice by targeting *Trpm*7 in early stages of oocyte and follicle development would be a major step forward to elucidate the function of *Trpm7* on oogenesis, fertilization and pre-implantation embryo development in mammals.

## Methods

### Oocyte and embryo collection

Six-to-twelve-week-old females (CD1, *Trpv3*^−/−^ colony^18^) were superovulated with intraperitoneal (i.p.) injection of 5 IU pregnant mare’s serum gonadotropin (PSMG, Sigma), followed 48 h later by i.p. injection of 5 IU of human chorionic gonadotropin (hCG, Sigma). Ovulated eggs (cumulus masses) were obtained by pulling the oviducts open with fine forceps in a HEPES-buffered culture medium (M2 medium, Millipore) 13–16 h after administration of hCG. In order to obtain 2 cell-stage embryos, females were mated with C57BL/6 males at the same time of the hCG injection. 2 cell-stage embryos were collected 40 h post hCG-injection. Cumulus cells were removed using hyaluronidase (Sigma) and gentle aspiration through a pipette. The zona pellucidae (ZP) were removed by exposure to Tyrode’s acid solution (pH 2.5) for a few seconds followed by thorough washing in M2 medium. Blastomers were separated mechanically by gentle aspiration through a pipette. GV oocytes were collected from the ovaries of 5- to 12-week-old *Trpv3*^−/−^ or CD-1 female mice. Females were injected with 5 IU PMSG and cumulus cell-enclosed oocytes were recovered 42–46 h later into Chatot, Ziomek, or Bavister (CZB) medium and 100 μM isobutyl-1-methylxanthine (IBMX). Experimental protocols completed at Aarhus University were performed according to the Danish national and Institutional regulations and approved by the Animal Experiments Inspectorate under the Danish Ministry of Justice (permit numbers 2015-15-0201-00800, 2012-15-2935-00002).

### Calcium imaging

[Ca^2+^]_i_ imaging was carried out as previously described^40^. In brief, [Ca^2+^]_i_ was measured using the Ca^2+^ sensitive dyes Fura-2-acetoxymethyl ester (Fura 2-AM, Molecular Probes; Invitrogen). Eggs were loaded with 1.25 μM Fura-2AM supplemented with 0.02% pluronic acid (Molecular Probes) for 20–30 min at room temperature. To estimate [Ca^2+^]_i_, oocytes/eggs were thoroughly washed and attached to glass-bottom chambers. For Fig. 1, responses to SrCl_2_ were recorded in FCS-free (serum free) HEPES-buffered Chatot, Ziomek, or Bavister (HCZB) medium, where external Ca^2+^ was replaced by 10 mM SrCl_2_, and under mineral oil. Ca^2+^ measurements were performed on the stage of an Olympus IX70 microscope equipped with an Olympus LUCPlanFL N × 20 objective (N.A. 0.45) and an EasyRatioPro fluorescence imaging system (Photon Technology International, NJ, USA). The oocytes were exited at 340 and 380 nm and emission was recorded at 510 nM. Intracellular Ca^2+^ ([Ca^2+^]_i_) was expressed from 340/380 fluorescence ratio. For Fig. 4, oocytes and eggs were recorded in FCS-free (serum free) TL-HEPES medium containing Ca^2+^ (2.04 mM), and Mg^2+^ (0.492 mM) under mineral oil. For Fig. 5, oocytes and eggs were recorded in FCS-free TL-HEPES containing the Ca^2+^ concentration described in the figure. For the Sr^2+^- induced oscillations measurements, Ca^2+^ was replaced by 10 mM SrCl_2._ Oocytes and eggs were monitored using inverted microscope (Nikon) fluorescence measurements. Fura 2-AM was excited between 340 nm and 380 nm by a filter wheel (Ludl Electronic Products Ltd.), and fluorescence was obtained every 20 s. After passing through a 510 nm, the emitted light was collected by a Cool Photometrics SenSys CCD camera (Roper Scientific, Tucson, AZ) to calculate fluorescence ratios of 340/380 nm in the whole egg.

### Electrophysiology

Whole-cell currents were measured at 22–24 °C using an HEKA 10 USB amplifier. Electrophysiology recordings were performed on the same day of oocyte, egg or embryo isolation up to 8 hours post-surgery. Eggs and embryo were maintained in human tubal fluid medium (HTF, EMD Millipore) and GV oocytes in CZB medium (EMD Millipore) at 37 °C and 5% CO_2_. Data were analyzed using Igor Pro and Origin 7.0 (OriginLab). Pipettes of 1–3 MΩ resistance were made from glass capillaries (593600, A-M systems). The intracellular solution contained (in mM): 142 Cs-Methanesulfonate, 10 HEPES, 3 NaATP, 0.3 NaGTP, 5 EGTA, 3 mM CaCl_2_ (free 100 nM) pH: 7.3–7.4. Concentration of Calcium was calculated using WincMax Chelator. The external solution for giga seal formation contained (in mM): 125 NaCl, 6 KCl, 20 mM CaCl_2_, 1.2 MgCl_2_, 20 mM HEPES-NaOH, pH: 7.3–7.4. TRPM7 basal responses to agonists or blockers, were measured in an external solution containing (in mM): 140 mM NaMES, 10 mM HEPES, 10 mM glucose, 4 mM KCl, and 2 mM CaCl_2_. The solution of NaMES was used in order to avoid chloride currents^24^. The osmolarity of all solutions was 290–320 mOsm. All voltages were corrected for calculated junction potentials present between the internal and external solution before seal formation. TRPM7 currents were activated by voltage ramps from 100 mV to −100 mV (600 ms, every 2 s), in the presence of the agonist or blockers. The holding potential (HP) was 0. Statistical analyses were performed using GraphPad Prism: t-test, paired, two-tailed P value (Figs 1, 3 and 7a) and One-way ANOVA (Fig. 7b).

### Chemicals

NS8593 (SIGMA), Naltriben (SIGMA).

### *In vitro* pre-implantation embryo development

Zygotes were collected from the oviducts of 6 to 8-weeks-old super ovulated CD1 and TRPV3KO females mated to CD1 males. CD1 zygotes were recovered 15 h post-hCG whereas TRPV3KO zygotes were collected at 20 h post-hCG, as earlier collection greatly reduced *in vitro* pre-implantation development even in control embryos. After collection, zygotes were cultured in KSOM media (Specialty Media) with or without 10 μM NS8593 in four-well dishes (Thermo Scientific Nunc, Fisher). Assessment of embryo development was made every 12 or 24 h according to the experimental design until 96 h of culture, where blastocysts rates were assessed. At least two replicates were performed per strain and Chi square tests were performed for significance.

## Additional Information

**How to cite this article**: Carvacho, I. *et al*. TRPM7-like channels are functionally expressed in oocytes and modulate post-fertilization embryo development in mouse. *Sci. Rep.* **6**, 34236; doi: 10.1038/srep34236 (2016).

## Supplementary Material

Supplementary Information

## Figures and Tables

**Table 1:** NS8593 inhibits pre-implantation embryo development to the blastocyst stage of mouse WT CD1 zygotes.

Treatment	#2^nd^ PB/2PN^a^	#2-Cells (%)^b^	#Blastocysts (%)^b^
Control	55	41 (75%)	27 (66%)
NS8593-10 μM	59	47 (80%)	1 (0.02%)

^a^Zygotes were collected 15 h post hCG. Four replicates, PB: Polar Body, PN: Pro-Nucleus.

^b^2-cell embryos were evaluated at 24 h and blastocysts at 96 h post collection, respectively.

**Table 2:** NS8593 delays developmental progression of mouse WT CD1 zygotes prior to the morula stage.

Treatment	#2^nd^ PB/2PN^a^	#2-Cells (%)^b^	#4-cells (%)	#8 -cells (%)
Control	32	26 (81)	26 (100)	21 (81)
NS8593-10 μM	35	31 (88)	21 (69)	6 (29)

^a^Zygotes were collected 15 h post hCG. Two replicates, PB: Polar Body, PN: Pro-Nucleus.

^b^2-cell embryos were evaluated at 24 h post collection, 4-cell embryos at 48 h and 8-cell embryos at 72 h.
