# Supplementary material for: TRPM7-like channels are functionally expressed in oocytes and modulate post-fertilization embryo development in mouse
Source: Sci Rep. 2016 Sep 29;6:34236. doi: 10.1038/srep34236 (PMC5041074; doi:10.1038/srep34236)
Supplement: Supplementary Information [file srep34236-s1.pdf]

TRPM7-like channels are functionally expressed in oocytes and modulate post-fertilization embryo development in mouse

Ingrid Carvacho[1,2,3*], Goli Ardestani[4], Hoi Chang Lee[4], Kaitlyn McGarvey[4], Rafael A. Fissore[4] and Karin Lykke-Hartmann[1,2,5*]

[1]Department of Biomedicine, Aarhus University, DK-8000 Aarhus C, Denmark.

[2]Centre for Membrane Pumps in Cells and Disease-PUMPKIN, Danish National Research Foundation, Aarhus University, Department of Molecular Biology and Genetics, DK-8000 Aarhus C, Denmark.

[3]Department of Biology and Chemistry, Faculty of Basic Sciences, Universidad Católica del Maule, 3480112 Talca, Chile.

[4]Department of Veterinary and Animal Sciences, University of Massachusetts, Amherst, MA 01003, USA.

[5]Aarhus Institute of Advanced Studies (AIAS), Aarhus University, DK-8000 Aarhus C, Denmark

* Co-corresponding authors: Ingrid Carvacho, icarvacho@biomed.au.dk, phone: +45-87167258, and Karin Lykke-Hartmann, kly@biomed.au.dk, phone: +45-29390558.

Supplementary Figure S1.

a. MII eggs

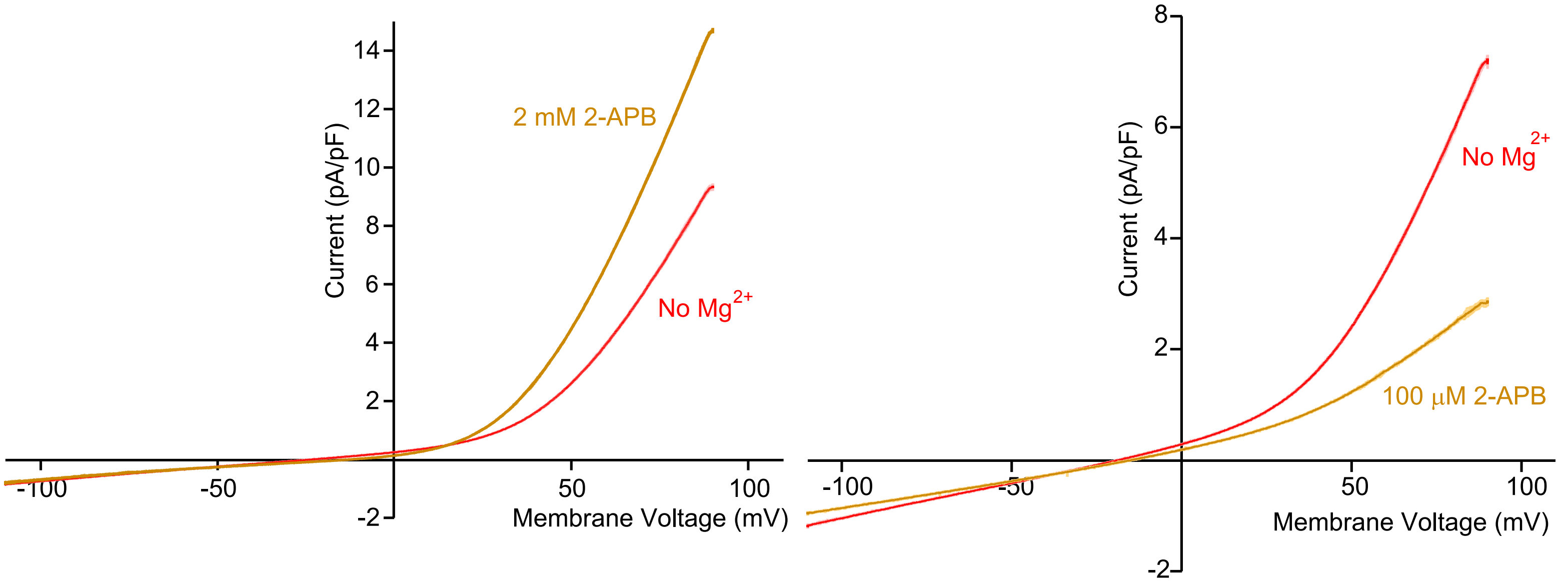

Figure S1. Modulation of native TRPM7 current by 2-APB in MII eggs. a. Whole-cell voltage clamp recordings from MII eggs. Current evoked from a voltage ramp from -100 to +100 mV. *Left panel*. 2-APB 2 mM increased the TRPM7-like current in MII eggs (n=5). *Right panel*. 2-APB 100 µM blocked TRPM7 current (n=4).

Supplementary Figure S2.

2-cell blastomeres

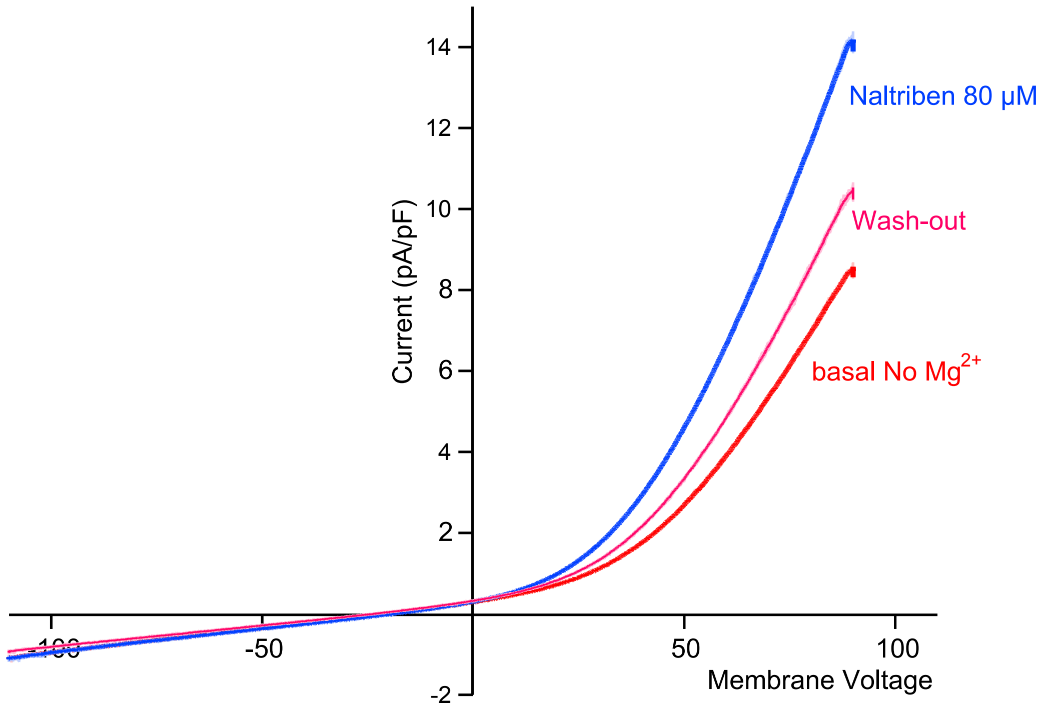

Figure S2. Naltriben potentiates TRPM7-like currents in 2-cell blastomeres. Whole-cell patch clamp recordings in blastomeres from 2-cell stage obtained after fertilization. Current evoked from a voltage ramp from -100 to +100 mV in basal conditions (red trace, basal No Mg^{2+}), presence of 80 μM Naltriben (blue trace) and after Naltriben wash-out (pink trace). n=3.

Supplementary Table S1. Apamin does not inhibit pre-implantation embryo development.

**Treatment**	**# of 2nd PB/2 PN**[a]	**# of 2 cell (%)**[b]	**# of 4 cell (%)**	**# of 8 cell (%)**	**# Blastocysts(%)**
Control	15	11 (73)	11 (100)	11 (100)	9 (81)
Apamin – 1 µM	26	17 (65)	17 (100)	16 (94)	14 (82)
Apamin – 10 uM	8	7 (88)	7 (100)	7 (100)	6 (86)

[a] Zygotes were collected 15 h post hCG. One replicate. PB: Polar Body, PN: Pro-Nucleus.

[b] 2-cell embryos were evaluated 24 h post-collection, 4-cell embryos at 48 h, 8-cell embryos at 72 h, and blastocysts at 96 h.

Supplementary Table S2. NS8593 delays pre-implantation embryo development to the blastocysts stage of mouse *Trpv3*$^{-/-}$ zygotes.

**Time of observation**		**24-h**	**48-h**	**60-h**		**72-h**		**96-h**
**Treatment**	# 2PN[a]	# 2-cells - (%)[b]	# 4-cells (%)	# 8-cells (%)	# Morula (%)	# Morula (%)	# Early Bl (%)	Blastocysts (%)
Control	22	22 (100)	22 (100)	11 (50)	11 (50)	12 (50)	10 (45)	22 (100%)
NS8593-10 μM	25	25 (100)	24 (96)	21 (88)	0 (0)	20 (95)	0 (0)	20 (80%)

[a] Zygotes were collected 20 h post hCG. Two replicates. PN: Pro-Nucleus.
[b] Time of observation to determine cleavage are indicated on top of each column.

Supplementary Table S3. **Waixenicin A negatively affects oocyte viability during *in-vitro* maturation.**

**Treatment**	# of GVs[a]	GVBD[b](%)	MII (%)
Control	35	33 (94.2%)	33 (94.2%)
Waixenicin A[c]	63	41 (65%)	0 (0.0%)

[a]GV oocytes were collected 48h post PMSG. Three replicates. [b]GVBD: Germinal Vesicle Break Down, MII: Metaphase II eggs. GVBD was evaluated 2 h post initiation of maturation and MII eggs were observed at 12 h post initiation of maturation. Oocytes in the treatment group that failed to undergo GVBD or reached the MII stage lysed and degenerated during culture. [c]Waixenicin A was used at 1 μM concentration, which is the minimal concentration required to prevent/reduce $[Ca^{2+}]_i$ oscillations in GV oocytes.
